# Treatment outcomes in cytomegalovirus anterior uveitis

**DOI:** 10.1038/s41598-024-66224-5

**Published:** 2024-07-02

**Authors:** Thanut Boonhaijaroen, Pitipol Choopong, Usanee Tungsattayathitthan, Nattaporn Tesavibul, Wilawan Sanphan, Sutasinee Boonsopon

**Affiliations:** Department of Ophthalmology, Faculty of Medicine, Siriraj Hospital, Mahidol University, Bangkok, Thailand

**Keywords:** Topical ganciclovir, Systemic ganciclovir, CMV anterior uveitis, CMV treatment, Diseases, Eye diseases, Uveal diseases

## Abstract

This retrospective cohort study investigated patients with cytomegalovirus anterior uveitis (CMV AU) and compared treatment outcomes between regional and systemic antiviral therapies. Treatment modalities included topical (2% ganciclovir [GCV] eye drops or 0.2% GCV eye gel) and systemic (intravenous GCV or oral valganciclovir) groups. The comparison parameters included response rates, time to response, recurrence rates, time to recurrence, and complications. Forty-four patients (54.5% male) with a mean age of 56 ± 9.87 years were enrolled, with 31 eyes in the topical group and 13 eyes in the systemic group. The median response time was significantly slower in the topical group (63 days [IQR 28–112]) compared to the systemic group (28 days [IQR 24–59]) (*p* = 0.04). Treatment response rates were 87.1% (27/31) in the topical group and 100% (13/13) in the systemic group (*p* = 0.30), while recurrence rates were 37% (10/27) and 69.2% (9/13) (*p* = 0.056), with a median time to recurrence of 483 days [IQR 145–1388] and 392 days [IQR 203.5–1907.5] (*p* = 0.20), respectively. In conclusion, both topical and systemic GCV treatments demonstrated favorable outcomes for CMV AU. Systemic GCV showed rapid control of intraocular inflammation.

## Introduction

Cytomegalovirus (CMV) anterior uveitis (AU) is the predominant malady arising from CMV infection among individuals with an intact immune system. The ocular pathophysiological mechanism underlying CMV infection involves the reactivation of a latent virus within the host's body. Epidemiological evidence reveals a higher incidence of CMV AU in Asian countries (ranging from 69.1 to 98.6%), in contrast to European nations (with rates of 41.9–57%). This divergence is attributed to disparate genetic factors across global populations and genetic variations inherent in the virus itself^1,2^. Typical manifestations of CMV infection include compromised visual acuity (VA), ocular discomfort, ipsilateral headache, and halos vision. Clinical signs align within three distinct categories: Posner–Schlossman syndrome (PSS), Fuchs uveitis syndrome (FUS), and corneal endotheliitis. PSS or glaucomatocyclitic crisis (GCC) is characterized by conjunctival injection, edematous corneal epithelium, and medium to large granulomatous keratic precipitates (KPs), occasionally exhibiting a gray-white hue. A mild anterior chamber (A/C) reaction of 0.5–1 + may accompany these features. Intraocular pressure (IOP) can reach 50 mmHg, although posterior synechiae may not be evident. While FUS shares similarities with PSS, it may also present with features such as heterochromia iridis or iris atrophy. Corneal endotheliitis, typified by inflammation of the corneal endothelial layer, can culminate in profound stromal corneal edema and KPs^1–3^.

The contemporary therapeutic approach to CMV AU focuses on antiviral agents, available through both topical and systemic routes. Conventional management entails a combination of topical or systemic ganciclovir (GCV) or valganciclovir, coupled with topical corticosteroids to mitigate A/C inflammation. Cycloplegic agents are prescribed to alleviate severe ocular pain and A/C reactions. Patients who have elevated IOP receive IOP-lowering agents to prevent future glaucomatous complications^2,4^. A study conducted in Singapore that examined the results of CMV treatment discerned favorable responses to systemic GCV, intravitreal GCV, and GCV implants. However, these modalities exhibited elevated recurrence rates. In particular, moderate response levels and lower recurrence rates were observed with the use of GCV gel as a treatment approach^5^. A comparison between the GCV gel group and nonusers highlighted diminished recurrence rates in the former^6^. Furthermore, studies conducted in Thailand and China evaluated the efficacy of 2% GCV eye drops in CMV treatment. The results suggested a feasible control of the infection for up to 12 months, with minimal side effects attributed to topical eye drops^7,8^. Complications of this disease include glaucoma, cataract formation, and corneal decompensation^1,2^. In the current landscape, no single CMV treatment has been universally endorsed as the primary therapeutic avenue. This is attributable to the presence of advantages and drawbacks inherent in both topical and systemic administration routes. As the superiority of outcomes in CMV treatment using systemic GCV versus topical GCV form remains unclear, we aim to assess the efficacy of both treatment modalities in CMV AU patients. This evaluation will be beneficial in identifying the most suitable treatment approach for patients in the future.

## Materials and methods

This retrospective cohort study was conducted at Siriraj Hospital between 2002 and 2022. This research was approved by the ethics committee of Siriraj Institutional Review Board, Faculty of Medicine, Siriraj Hospital, Mahidol University, Thailand (Si266/2022) following the principles of the Declaration of Helsinki, and waived the requirement for informed consent forms. This study was registered in the Thai Clinical Trials Registry, https://www.thaiclinicaltrials.org/show/TCTR20230401002 (reg no. TCTR 20230401002, registered date 01/04/2023). The study comprised patients aged 18 and older, all of whom were immunocompetent and had received a definitive diagnosis of CMV AU. The confirmation of CMV AU was established through aqueous polymerase chain reaction testing. The initial treatment approach for the patient involves the use of exclusively topical GCV eye drops/ eye gel or systemic GCV, without combination. Patients exhibiting CMV ocular infection in a location other than the anterior segment were excluded from the study. Treatment approaches were classified into two groups: the topical group (comprising 2% GCV eye drops or 0.15–0.2% GCV gel) and the systemic group (involving intravenous GCV or oral valganciclovir). The medication available during the study period consisted of in-house 2% GCV eye drops formulated in a balanced salt solution, in-house 0.2% GCV eye gel, 0.15% commercially available GCV eye gel (Virgan^®^), oral valganciclovir tablet, and GCV infusion.

The primary outcome measure was the treatment response rate. Secondary outcomes included response time, recurrence rate, time to recurrence, VA improvement, IOP improvement, and complications. Baseline clinical characteristics and adverse events resulting from treatments were documented. The treatment response was defined by reduction in A/C cells to a level below 1 + and absence of active KPs. Topical steroids eye drops should not exceed 4 drops per day, with a concentration of 1% prednisolone acetate or lower potency. Recurrence was identified by the presence of A/C cells that exceed a level of 1 +, and new occurrences of KPs. In cases of treatment failure, it was determined by the inability to reduce A/C cells by more than 2 steps or the persistence of A/C cells above level 1 +, along with the persistence of active KPs. Time to response referred to the duration between the initiation of treatment and the documentation of A/C cells and KP that correspond to the definition of treatment response, while time to recurrence was defined as the duration between a successful treatment response and the subsequent new onset of intraocular inflammation. A/C cells grading adhered to the guidelines established by the Standardization of Uveitis Nomenclature (SUN) Working Group^9^.

### Statistical analysis

The sample size calculation used the formula to compare two independent proportions. For the present study, the values of the systemic group and the topical group were established at 0.9 and 0.6^5^, respectively, with a significance level of 0.05 and a statistical power of 80%. The resulting calculation yielded a minimum sample size of 75 participants. Statistical analysis was performed using PASW Statistic (SPSS) 23.0 (SPSS, Inc., Chicago, IL, USA). Descriptive statistics included frequency and percentage for categorical variables, while continuous variables were expressed as means and standard deviations for normally distributed variables and as medians (Percentile 25 and Percentile 75) for nonnormally distributed variables. The mean comparisons of continuous variables between groups used the Student's t test and the Mann–Whitney test, while the comparisons of categorical variables used the Chi-square test or Fisher’s exact test. Kaplan–Meier curve analysis and the logarithmic rank test assessed time to response and time to recurrence. Throughout all tests, a two-tailed *p*-value < 0.05 was considered statistically significant.

## Results

**Figure 1:**
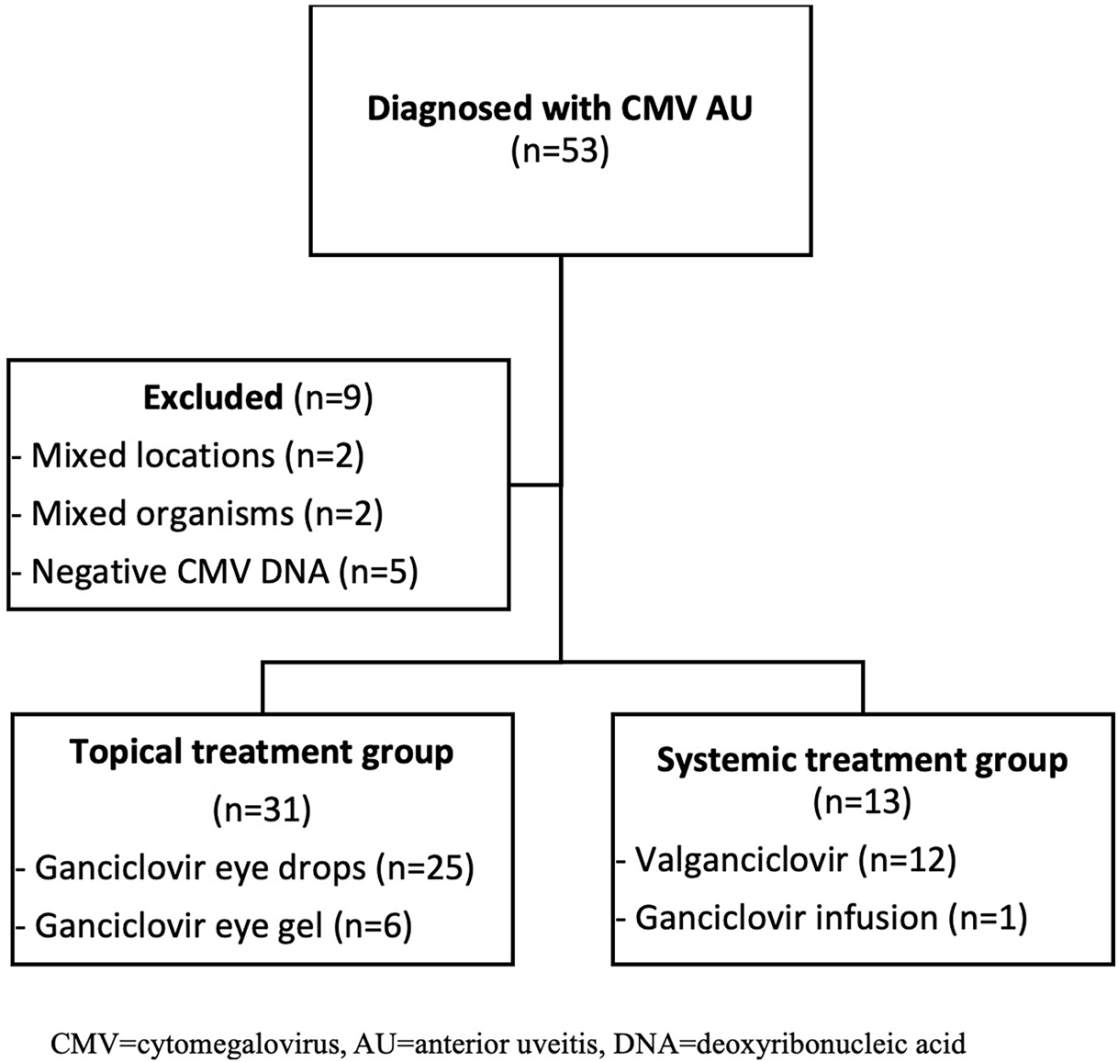
CONSORT Flow Diagram. Flow diagram illustrating the patient selection process for the study on cytomegalovirus anterior uveitis (CMV AU). Fifty-three patients initially diagnosed with CMV AU were identified. Nine patients were excluded due to mixed locations of inflammation, mixed organisms, or negative CMV DNA. The remaining 44 patients were allocated into two treatment groups: topical treatment (n = 31) and systemic treatment (n = 13).

A total of 53 patients diagnosed with CMV AU were initially included in the study. However, 9 of these patients were later excluded: 2 patients had inflammation in more than one location (one patient had anterior uveitis plus intermediate uveitis and another had panuveitis), 2 patients had positive DNA from mixed organisms, and 5 patients had negative CMV DNA from aqueous analysis. The CONSORT flow diagram is shown in Fig. 1. The remaining forty-four patients (54.5% male), with an average age of 56 ± 9.87 years, were enrolled in the study. Most of the patients (93.2%) had GCC, with a median initial IOP of 23.5 mmHg (IQR 16–35). There were 31 eyes in the topical group and 13 eyes in the systemic group. We found no discernible differences in initial VA and IOP between the two groups, as shown in Table 1. It is worth mentioning that a majority of patients with normal IOP were already receiving antiglaucoma medication.

**Table 1:** Baseline characteristics of all patients and comparison between those treated with topical ganciclovir and systemic ganciclovir.

Parameters	Total patients N = 44	Topical group n = 31	Systemic group n = 13	*P*-value
Age (years), mean ± SD	56 ± 9.87	56 ± 10.71	55 ± 7.81	0.74
Male, n(%)	24 (54.5)	17 (54.8)	7 (53.8)	0.95
Presentation				
GCC, n(%)	41 (93.2)	29 (93.5)	12 (92.3)	1
FUS, n(%)	3 (6.8)	2 (6.5)	1 (7.7)	
Initial VA, median (IQR)	6/7.5 (6/7.5–6/15)	6/7.5 (6/6–6/18)	6/7.5 (6/6–6/9.5)	0.21
Initial IOP (mmHg), median (IQR)	23.5 (16–35)	20 (14–36)	28 (19.5–35.5)	0.38

*GCC* glaucomatocyclitic crisis, *FUS* Fuch’s uveitis syndrome, *VA* visual acuity, *IOP* intraocular pressure.

A *p*-value < 0.05 indicates statistical significance.

Upon diagnosis, patients in the topical group were initially treated with 2% GCV eye drops eight times a day (n = 25). Alternatively, 0.15–0.2% GCV eye gel was administered five to eight times daily (n = 6), with subsequent adjustments based on disease activity. In the systemic group, patients received 450–900 mg of valganciclovir twice daily for four weeks, followed by a reduction to 450 mg twice daily from four to twenty weeks (n = 12). Only one patient was treated with intravenous GCV infusion at 5 mg/kg twice daily for 14 days, followed by 900 mg of oral valganciclovir twice daily for four weeks. The mean duration of systemic therapy was 2.42 ± 1.85 months, while the mean duration of topical therapy was 43.93 ± 25.38 months. Throughout the study, complete blood counts and serum creatinine levels were monitored every four weeks for the systemic treatment group. Additionally, to manage anterior segment inflammation, 1% prednisolone acetate eye drops were provided. Dosages were individually tailored on the basis of A/C reactions. Both topical and systemic antiglaucoma drugs were used to regulate IOP. The topical group showed treatment response rates of 87.1% (27/31), while the systemic group demonstrated a 100% response rate (13/13), resulting in a non-significant statistical difference (*p* = 0.30). Furthermore, the recurrence rates between the two groups did not show significant differences: 37% (10/27) in the topical treatment group and 69.2% (9/13) in the systemic treatment group (*p* = 0.056), as illustrated in Table 2.

**Table 2:** Treatment results and complications compared between the topical treatment group and the systemic treatment group.

Parameters	Topical group	Systemic group	*P*-value
Treatment response, n(%)			
Response	27 (87.1)	13 (100.0)	0.30
Response with remission	17 (63.0)	4 (30.8)	
Response with recurrence	10 (37.0)	9 (69.2)	
Failure	4 (12.9)	0	
VA improvement, median (IQR)	0 (6/4.8–6/7.5)	0 (6/4–6/7.5)	0.29
IOP improvement, median (IQR)	8 (0–23)	10 (5.5–21)	0.34
Complications, n(%)			
Corneal decompensation	2 (6.5)	1 (7.7)	1
Secondary ocular hypertension	21 (67.7)	10 (76.9)	0.72

*VA* visual acuity, *IOP* intraocular pressure.

A *p*-value < 0.05 indicates statistical significance.

**Figure 2:**
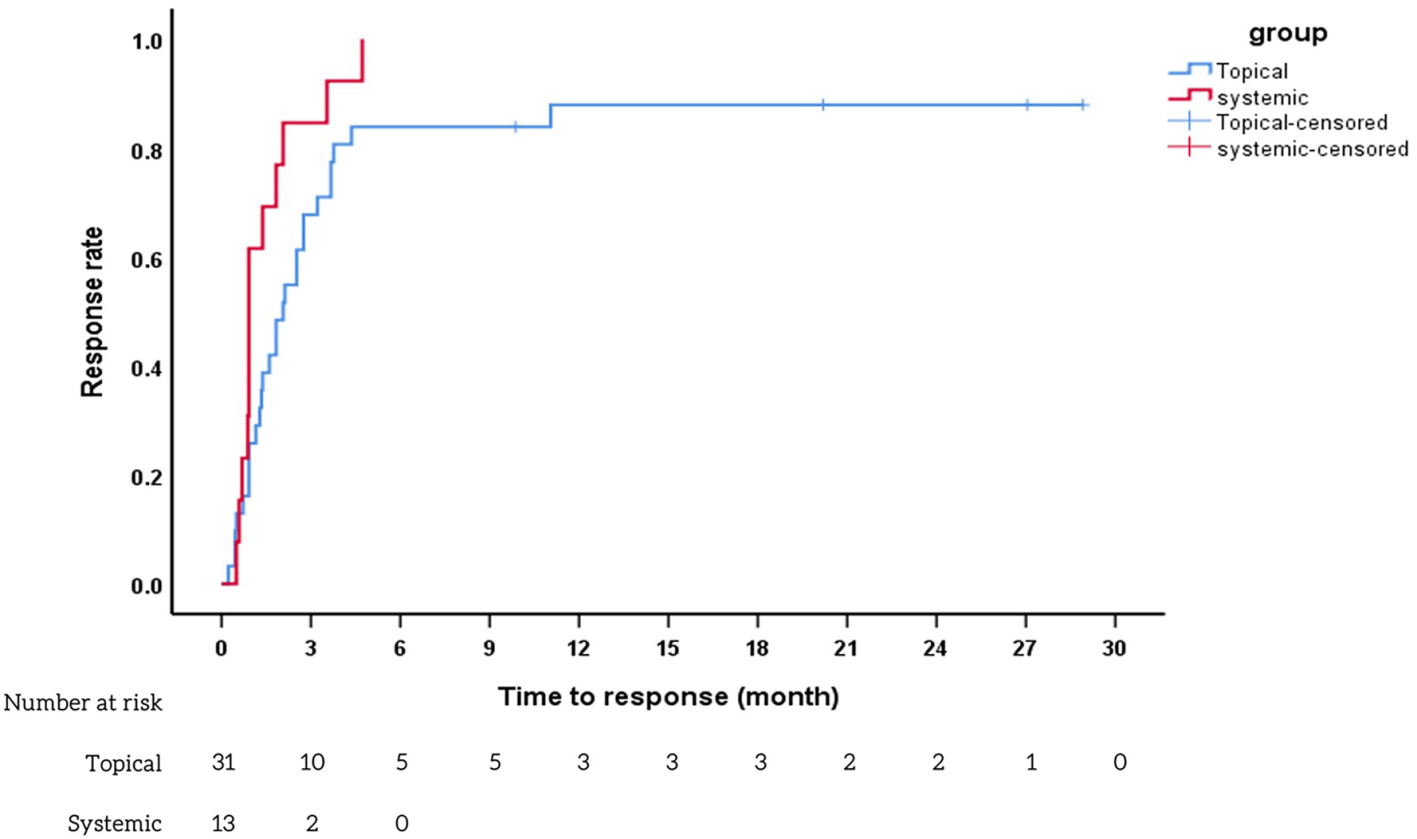
Kaplan–Meier survival analysis to compare response time between topical and systemic groups. Kaplan–Meier survival curves illustrating the treatment response rates in patients with cytomegalovirus anterior uveitis treated with systemic therapy (red line) compared to topical therapy (blue line). The red line demonstrates a rapid treatment response rate in the systemic therapy group, whereas the blue line represents the response rate in the topical therapy group.

**Figure 3:**
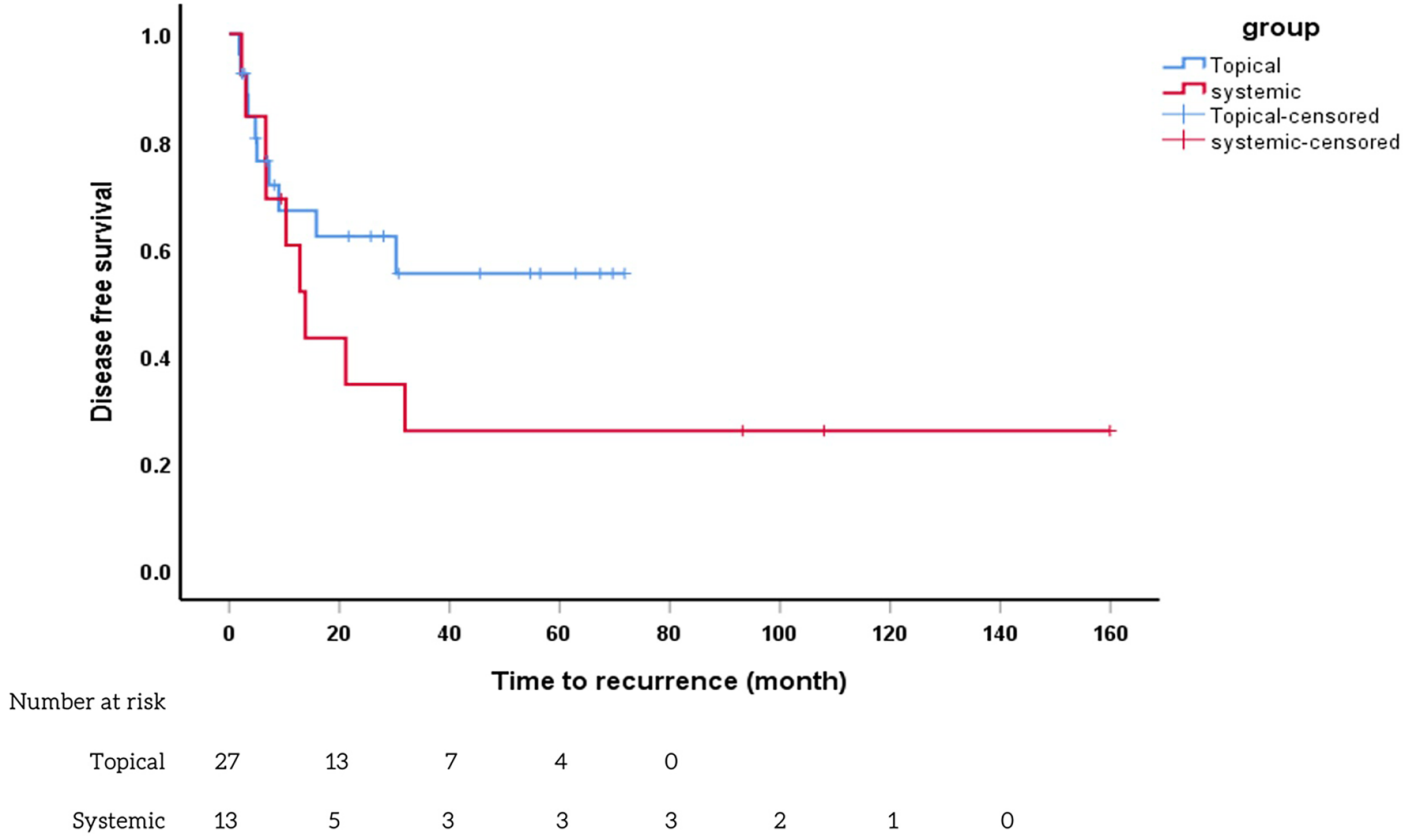
Kaplan–Meier survival analysis to compare the time to recurrence between the topical and systemic groups. Kaplan–Meier curves depicting the time to recurrence of cytomegalovirus anterior uveitis in patients treated with systemic therapy (red line) and topical therapy (blue line). Both groups showed recurrent inflammation at approximately 13 months (systemic therapy) and 16 months (topical therapy), with no statistically significant difference observed (*p* > 0.05).

The median response time was significantly longer in the topical group compared to the systemic group (63 days [IQR 28–112] and 28 days [IQR 24–59], respectively; *p* = 0.04), as illustrated in Fig. 2. Regarding the time to recurrence, the median durations were 483 days (IQR 145–1388) in the topical group and 392 days (IQR 203.5–1907.5) in the systemic group (*p* = 0.20), as shown in Fig. 3. Secondary ocular hypertension (OHT), defined as an IOP exceeding 21 mmHg, was observed in 67.7% (21/31) of the topical group and 76.9% (10/13) of the systemic group. This complication appeared more frequently compared to corneal decompensation, which was found in 6.5% (2/31) of the topical group and 7.7% (1/13) of the systemic group, respectively. Trabeculectomy was performed in 38.1% (8/21) of the topical group and 10% (1/10) of the systemic group. Only one patient in the systemic group underwent penetrating keratoplasty. In terms of IOP control, both groups experienced a decrease from baseline (8 mmHg in the topical group and 10 mmHg in the systemic group). No significant differences in VA were observed between pretreatment and posttreatment measurements in either group, as indicated in Table 2. Only 12.9% (4/31) of the eyes in the topical group attempted to discontinue topical medication, while a notable 84.6% (11/13) of the eyes in the systemic group made such attempts. No adverse events were reported in the topical group, while a single event of reversible neutropenia resulting from oral valganciclovir was documented in the systemic group. The mean duration of follow-up was 64.45 ± 31.75 months for the topical group and 108 ± 30.39 months for the systemic group. The follow-up duration for the topical group was significantly shorter compared to the systemic group, with a *p*-value of 0.0001.

## Discussion

CMV AU presents a formidable challenge in terms of management, where the selection of an appropriate treatment modality assumes a pivotal role in the achievement of favorable outcomes. Within the context of this retrospective cohort study, we performed a comparative analysis of the treatment outcomes for CMV AU, comparing regional antiviral therapy against systemic antiviral medication within the confines of the Thai population for two decades. Our study disclosed that both treatment modalities showed commendable rates of treatment response. Accorinti et al. found comparable results with no statistically significant differences between topical therapy alone and a combination of topical and systemic medication. It should be noted that they administered oral acyclovir in certain cases^10^. Interestingly, according to TITAN report 2, most experts worldwide (over 70%) prefer to begin treatment for CMV AU with topical antivirals^11^. The cost and potential side effects associated with systemic antiviral medication are the main reasons to consider its use only when necessary. In contrast, Chee et al. observed that patients receiving systemic GCV, intravitreal GCV injection, and GCV implant exhibited a good initial response. However, the response to GCV eye gel was moderate with unpredictable outcomes^5^. The key difference between their study and ours was the frequency of topical drug administration. For our own GCV eye drops and eye gel, we generally started the medication at a frequency as high as every 2 h until night. For the commercially available GCV eye gel, our prescription regimen involved administering it 5 times a day. However, a study conducted in Singapore opted for a dose of 4 times a day to alleviate the burden on patients^5^. We believe that variation in drug frequency could potentially affect treatment results. However, we observed a statistically significant advantage of systemic GCV over topical medications in terms of achieving a rapid treatment response, roughly within 1 month compared to 2 months, respectively. Based on these results, systemic GCV may be considered as the first-line treatment in cases presenting severe inflammation, as it can rapidly control ocular inflammation. As widely understood, GCV is a virostatic drug, which means that it does not permanently eliminate the virus^12^. Hence, recurrent infection is common. Preventing recurrent inflammation is crucial to avoid irreversible complications, specifically secondary glaucoma and corneal decompensation. This underscores the importance of investigating which route of drug administration can effectively prevent further ocular damage through long-term infection control. In our study, we did not observe a difference in recurrence rates between the two groups. Given previous reports suggesting that systemic GCV may be less effective in preventing recurrent inflammation compared to topical GCV, further investigation with a larger study population is warranted to confirm our findings^5,6^. In our study, we observed an unfair comparison between the groups, as most patients in the topical group continued using their medication, with fewer attempts to discontinue it, while most of the systemic group sought to discontinue the medication. Notably, our study discerned a lack of noticeable changes in VA within both groups. Since most patients started treatment with good VA, it was difficult to improve VA beyond their initial levels after treatment. Notably, our study did not document cataract progression; however, we can speculate that cataracts might not be a significant issue among our study patients. The most common complication found in this study was secondary OHT. A congruent decrease of 8 to 10 mmHg was observed from baseline in both groups, in conjunction with the concurrent administration of IOP lowering medications. These results are comparable to previous studies^13,14^. Only 29% of our patients required surgical intervention. In other studies, a range of 25.7–60% of patients required glaucoma surgery^10,14,15^. We hypothesize that the initial aggressive use of high-frequency topical steroids in our clinic may contribute to rapid control of trabeculitis and IOP. Typically, we prescribe primarily 1% prednisolone acetate, administered up to every 2 h, especially if patients present with 2 + or more anterior chamber cells. Touhami et al. suggested that the early start of antiviral therapy (within or before 700 days) appeared to decrease the need for glaucoma surgery^15^.

Fortunately, neither treatment modality produced serious adverse events. It is particularly noteworthy that there were no events such as ocular irritation, ocular redness, and allergic conjunctivitis within the topical group, affirming the safety of employing topical antiviral agents for the treatment of AU. Numerous other studies have likewise demonstrated the efficacy of topical antiviral agents in the context of CMV AU^6,7,13^. The occurrence of neutropenia in a single patient receiving oral valganciclovir, although infrequent, highlights the importance of regular monitoring of complete blood counts.

### Limitations

This was a retrospective study. The total count of patients included in this study was relatively modest. The allocation of treatments to individual patients was done in a nonrandom manner. The follow-up duration differed markedly, with the topical group having a shorter duration compared to the systemic group. This difference may influence outcomes such as relapse rates. The inevitability of interference from topical steroid eye drops is acknowledged. Furthermore, the scope of parameters available for analysis was constrained. It is recommended that future investigations consider the sample size by enrolling a greater number of patients and contemplate the implementation of a randomized controlled trial methodology for more robust results.

## Conclusions

Treatment of CMV AU with topical GCV or systemic GCV showed favorable outcomes. A systemic GCV demonstrated a more rapid control of intraocular inflammation, making it a valuable option for initial treatment. To address the recurrent nature of CMV AU, prolonged antiviral prophylaxis may be necessary. Considering its good tolerability and minimal side effects, the ongoing use of topical GCV can be considered as a long-term maintenance therapy to prevent recurrent inflammation.

## Data Availability

The datasets generated and/or analyzed during the current study are available from the corresponding author on reasonable request.
